# FROM BEING ACTIVATED TO SELF-ORGANIZATION IN ELDER CARE: CONCEPTS OF AGING AND THEIR IMPACT ON RESIDENTS’ AGENCY

**DOI:** 10.1093/geroni/igz038.1872

**Published:** 2019-11-08

**Authors:** Irene Strasser, Ines Hopfgartner, Carmen Payer

**Affiliations:** 1 The American University of Paris, Paris, France; 2 University of Klagenfurt, Klagenfurt, Austria

## Abstract

In a research project together with an elder care facility in Austria, we were following a participatory approach to investigate important factors and processes to increase quality of life for residents with dementia. The aim was to better understand how we can foster participation, agency and freedom of choice within a care setting. Together with care workers, residents and relatives we were working on how to identify overall strategies and aims, as well as particular processes and procedures that allow for a higher involvement of all these groups of individuals. Doing research explicitly together with care workers we also aimed to trigger reflection of individual and organizational concepts of aging. We interviewed residents, relatives and care workers, conducted research workshops, and engaged in participant observation. In the talk the focus will be on care workers’ perspectives. We wanted to better understand the multiple stressors within daily routines in care work. Particularly, we wanted to find out more about the multifaceted processes of integrating one’s experiences into everyday care work, and integrating care work into one’s life story, to make meaning of the important work they are providing under constantly stressful working conditions. Finally, together with care workers, we developed a model for participation, that is explaining a wide range of residents’ possibilities for involvement: From activity orientation to actual participation and the realization of individually meaningful activities. What helps to initialize participation, and what we identified as obstacles in supporting residents’ autonomy will also be discussed in the talk.

